# Boosting with stumps for predicting transcription start sites

**DOI:** 10.1186/gb-2007-8-2-r17

**Published:** 2007-02-02

**Authors:** Xiaoyue Zhao, Zhenyu Xuan, Michael Q Zhang

**Affiliations:** 1 Cold Spring Harbor Laboratory, 1 Bungtown Road, Cold Spring Harbor, New York 11724, USA

## Abstract

Promoter prediction is a difficult but important problem in gene finding, and it is critical for elucidating the regulation of gene expression. We introduce a new promoter prediction program, CoreBoost, which applies a boosting technique with stumps to select important small-scale as well as large-scale features. CoreBoost improves greatly on locating transcription start sites. We also demonstrate that by further utilizing some tissue-specific information, better accuracy can be achieved.

## Abstract 2

CoreBoost applies a boosting technique to select important features for predicting core promoters with diverse patterns.

## Background

Initiation of transcription of protein coding genes is a very important step in the regulation of gene expression. This process starts with the assembly of the RNA polymerase II (Pol II) preinitiation complex at the promoter. The term 'promoter' commonly refers to the DNA region that is immediately upstream of a gene and that is required to control and regulate the transcription initiation of the gene. A core promoter with a length of about 100 base pairs (bp) is centered around the transcription start site (TSS), and a proximal promoter contains several hundred bases immediately upstream of the core promoter. The main characteristic of a promoter is that it contains clusters of transcription factor binding sites (TFBSs), which orchestrate the on-off switches of the target genes. Determining the location of the TSS is a critical step in identifying the promoter region, the study of which is necessary to elucidate gene expression patterns, regulatory networks, cell differentiation, and development.

Recently developed experimental methods such as 5'-end serial analysis of gene expression or cap analysis of gene expression, 5'-oligo capping technology, and chromatin immunoprecipitation (ChIP) followed by microarray hybridization (ChIP-chip) permit high-throughput profiling of TSSs [1-4]. However, there are many situations in which full-length cDNA information is not available; examples include novel genes and genes that are expressed at low levels. There is also much less information for many model organisms. Furthermore, computational methods may provide useful insights that suggest underlying biological mechanisms and correct systematic bias associated with certain experimental data. Therefore, *in silico *methods are useful, and there is demand for means to improve prediction accuracy.

The *in silico *identification of the 5' end of genes has been a challenging problem. A two-step approach to promoter recognition and TSS mapping has been proposed [5,6]: initial identification of a functional promoter in a roughly 2-kilobase (kb) region and further prediction of a TSS in a 50 bp region. The first step is on a larger scale, in which coarse-grained measures such as CpG islands, nucleosome binding, chromatin modification, downstream coding propensity, and transcription factor (TF) density are very useful. The second step is on a finer scale that needs more detailed features to best discriminate the precise TSS region from its surroundings. Recent advances in experimental technologies provide an ideal situation to revisit this two-step strategy. For example, results from Pol II ChIP-chip analysis can help us to focus the search. A core promoter prediction program can be subsequently used to map the TSS finely. With progress in both experimental and computational technologies, the accuracy and resolution of TSS predictions can be further improved by combining these complementary methods.

Many computational methods to predict promoters have been proposed. The underlying principle of these methods is that promoter regions have some characteristic features that make them distinct from nonpromoters. Predictive models using these features to discriminate promoters from nonpromoters are built and then used to search for new promoters in an input DNA sequence. Many of these methods are reviewed and compared in several recent reports [7-10]. Although there has been much success in locating the TSSs for CpG-related promoters, the performance for non-CpG-related promoters (about 25% of known genes) is still not satisfactory because of the diverse nature of vertebrate promoter sequences. To improve accuracy on both sets, especially on non-CpG-related promoters, is the goal of the present work. Recent studies demonstrate that it is computationally useful and biologically meaningful to treat CpG-related promoters and non-CpG-related promoters separately [11-13]. Choosing a characteristic set of biological signals specific for CpG and non-CpG-related promoters and applying appropriate algorithms for classification are very important for further improvement. To address this issue, we select features among position-specific core promoter elements, TFBSs, mechanical properties, and Markovian scores, as well as *k*-mer frequencies, and apply the LogitBoost procedure [14] with stumps (decision trees with two terminal nodes). Boosting sequentially applies a classifier to re-weighted training data, adding more weights to previously misclassified samples. It works well for the set of promoter sequences with diverse patterns.

This report is organized as follows. We first describe the characteristics of promoter DNA and explain the features used in CoreBoost. We then report the performance on different test data, including sequences from ChIP-chip experiments, and discuss challenges and future research directions. Finally, the LogitBoost algorithm and our proposal for using two binary classifiers for multiclass classification are presented.

## Results

### Feature profiles of promoter DNA

Eukaryotic Pol II promoters are controlled by a combination of core promoter elements, additional proximal upstream promoter elements, and enhancer elements. The core promoter spans -50 to +50 around the TSS and is essential for initiating basal transcription. There are only a few known core promoter elements. The TATA box and Inr are two key components, each of which can direct accurate transcription initiation by Pol II. The downstream promoter element, TFIIB recognition element, motif ten element, and downstream core element are some recently discovered core promoter elements [15,16]. However, not every element mentioned above occurs in a core promoter, which is an indication that there are more promoter features that are capable of mediating transcription. The proximal promoter region and distal enhancers contain multiple TFBSs that are responsible for transcriptional regulation [17]. The content and arrangement of these binding sites, together with local TF concentration, determine when and where the promoter is activated. It appears that the region from the TSS to approximately 250 bp upstream of the TSS is more enriched with TFBS [18]. This region might contain sites that are bound by activators or mediators, directing the formation of the preinitiation complex to the core promoter region. We took the region [-250, +50] as positive training data, and its immediate upstream and downstream as negative training data, and scanned for TFBSs. Those binding sites with higher binding affinity in promoter sequences than in non-promoter sequences are potentially useful for discrimination. (We use weight matrix to represent a TF as well as its binding motif, and use log likelihood ratio scores to measure the binding affinity of a site [19].)

**Figure 1:**
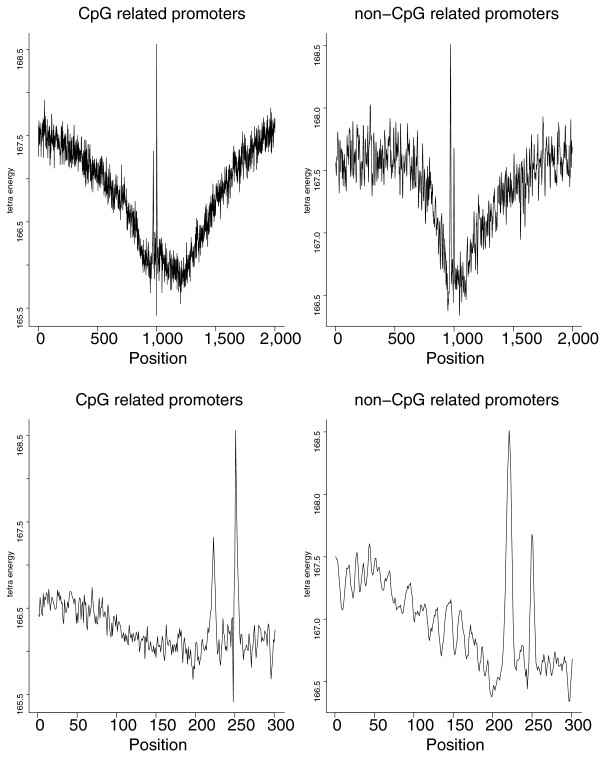
The energy profiles of CpG-related promoters and non-CpG-related promoters. The transcription start site is located at position 1000 in the top figures and position 250 in the bottom figures. All of the plots were smoothed with an average window of width 5. TSS, transcription start site.

The complexity and heterogeneity of promoters make it difficult to predict promoters *in silico*. Not all of the core promoter elements are consistently shared by Pol II promoters, and so they cannot discriminate promoters well by themselves. Based on a statistical analysis of the Eukaryotic Promoter Database (EPD) database, only 22% of the promoters have a TATA signal and only 49% have an Inr site [20]. However, recent studies show that TATA-less or Inr-less promoters have distinctive mechanical properties that are similar to TATA box or Inr containing promoters, which may function as markers for promoter recognition [21]. Figure 1 displays profiles of the negative of minimum energy for CpG-related (left) promoters and non-CpG-related (right) promoters. These profiles are based on tetranucleotide parameters, which are calculated from a database of tetranucleotide X-ray crystal structures and used to describe the potential energy of tetranucleotide sequences [22]. We see that for both CpG-related and non-CpG-related promoters, the regions around the TSS have much lower scores. There is also a sharp peak around positions -25 and +1, where TATA box and Inr are usually located. Similar plots (not shown) are also observed for profiles based on tetranucleotide flexibility parameters. DNA flexibility may play a role in the interaction between proteins and DNA. The regions surrounding the TSS tend to be more flexible, and the small segments around -25 and +1 more rigid. These distinctive energy and flexibility scores are also included in our feature set for promoter prediction.

Table 1 gives all of the feature types used to train CoreBoost. Table 2 lists the top features for the four binary classifiers (promoter against upstream and promoter against downstream for CpG-related and non-CpG-related promoters). (LogitBoost with stumps iteratively picks one feature at a time to minimize the current weighted loss function. The top features listed here are based on their selection order by the algorithm.) Our feature set contains binding affinities of core promoter elements and TFBS, tetranucleotide flexibility and energy scores, Markovian scores, and *k*-mer frequencies. Position-specific signals such as TATA box or Inr alone do not have much predictive power. Combining these with the signals from flanking sequences such as the flexibility/Markovian scores gives the best indication of the presence of a TSS. Detailed information about how these features are calculated is described under Materials and methods (below).

### Comparison with other promoter prediction methods

In this section we demonstrate the effectiveness of CoreBoost by comparing its performance with those of McPromoter [23] and Eponine [24], two of the best promoter predictors that are also freely and publicly available [9]. McPromoter uses neural networks to predict promoters by combining information from different segments (upstream, core promoter [TATA, spacer, and Inr] and downstream) and some structural features. Eponine applies a relevance vector machine with an optimal set of positioned weight matrices selected using a Monte Carlo process.

With CoreBoost sliding a window along a sequence, a vector of class probabilities is output. Positions with probability exceeding some threshold are considered possible candidates. Candidates within some distance are then clustered and the one with the best score in the cluster is output as the putative TSS. If a prediction is within 50 bp of an annotated TSS, then we call it a true positive hit. A threshold is chosen to achieve the desired sensitivity and positive predictive value (PPV). Sensitivity and PPV are defined under Materials and methods (below).

#### Comparison using ChIP-chip data

ChIP-chip data provide an opportunity to study the genome-wide map of active promoters in specific cells. Using this technology, the binding sites of preinitiation complex were experimentally located throughout the genome in human fibroblast cells [4]. Although TSSs cannot be located precisely from this experiment, a core promoter prediction program can be used subsequently to search for it. To evaluate the performance of CoreBoost, we applied different programs to the test sequences of 2.4 kb each centered at the predictions from the genome-wide mapping data of promoters. Each of these test sequences contain one and only one DataBase of Transcription Start Sites (DBTSS)-annotated TSS, which is used to count true positives.

**Figure 2:**
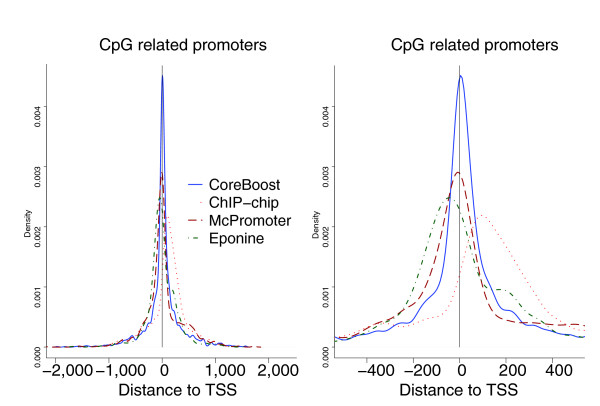
Density plot of the relative distance from the positions with maximal scores to the annotated TSS. The dot curve is based on the prediction from the ChIP-chip experiment. The solid curve is for CoreBoost. The dashed and the dot-dashed curves correspond to McPromoter and Eponine, respectively. The right figure is a zoomed in version of the left one. ChIP, chromatin immunoprecipitation; TSS, transcription start site.

For CpG-related predictions, we trained CoreBoost based on 1,445 promoters from EPD. Figure 2 shows a density plot of the relative distance from positions corresponding to maximal scores to the DBTSS-annotated TSS for all 1,765 CpG-related test promoters. If there were multiple positions corresponding to a maximal score, then a random one was picked. CoreBoost has 39% maximal scores achieved within 50 bp of an annotated TSS, which is significantly greater than McPromoter's 30% and Eponine's 23%. (Given a threshold, Eponine predicts a region with a maximal score associated. To locate the position with the maximal score for a test sequence, we run Eponine with a threshold equal to that score and take the middle position of the predicted region as the maximal position.) The corresponding *P *values based on one-sided *t*-test are 8 × 10^-9 ^and 0 respectively. Note that the *P *values are calculated using the following *t*-statistics:

T1=(0.39−0.3)/0.39×(1−0.39)1765+0.3×(1−0.3)1765T2=(0.39−0.23)/0.39×(1−0.39)1765+0.23×(1−0.23)1765.
 MathType@MTEF@5@5@+=feaafiart1ev1aaatCvAUfeBSjuyZL2yd9gzLbvyNv2Caerbhv2BYDwAHbqedmvETj2BSbqee0evGueE0jxyaibaiKI8=vI8tuQ8FMI8Gi=hEeeu0xXdbba9frFj0=OqFfea0dXdd9vqai=hGuQ8kuc9pgc9s8qqaq=dirpe0xb9q8qiLsFr0=vr0=vr0dc8meaabaqaciGacaGaaeqabaqadeqadaaakeaafaqabeGabaaabaGaamivamaaBaaaleaacaaIXaaabeaakiabg2da9iaacIcacaaIWaGaaiOlaiaaiodacaaI5aGaeyOeI0IaaGimaiaac6cacaaIZaGaaiykaiaac+cadaGcaaqaamaalaaabaGaaGimaiaac6cacaaIZaGaaGyoaiabgEna0kaacIcacaaIXaGaeyOeI0IaaGimaiaac6cacaaIZaGaaGyoaiaacMcaaeaacaaIXaGaaG4naiaaiAdacaaI1aaaaiabgUcaRmaalaaabaGaaGimaiaac6cacaaIZaGaey41aqRaaiikaiaaigdacqGHsislcaaIWaGaaiOlaiaaiodacaGGPaaabaGaaGymaiaaiEdacaaI2aGaaGynaaaaaSqabaaakeaacaWGubWaaSbaaSqaaiaaikdaaeqaaOGaeyypa0JaaiikaiaaicdacaGGUaGaaG4maiaaiMdacqGHsislcaaIWaGaaiOlaiaaikdacaaIZaGaaiykaiaac+cadaGcaaqaamaalaaabaGaaGimaiaac6cacaaIZaGaaGyoaiabgEna0kaacIcacaaIXaGaeyOeI0IaaGimaiaac6cacaaIZaGaaGyoaiaacMcaaeaacaaIXaGaaG4naiaaiAdacaaI1aaaaiabgUcaRmaalaaabaGaaGimaiaac6cacaaIYaGaaG4maiabgEna0kaacIcacaaIXaGaeyOeI0IaaGimaiaac6cacaaIYaGaaG4maiaacMcaaeaacaaIXaGaaG4naiaaiAdacaaI1aaaaaWcbeaakiaac6caaaaaaa@82CB@

There is a systematic downstream bias in the ChIP-chip data, which may be caused by the pausing of the polymerase at the downstream nucleosome. CoreBoost can be used in combination with ChIP-chip data to correct such bias.

**Figure 3:**
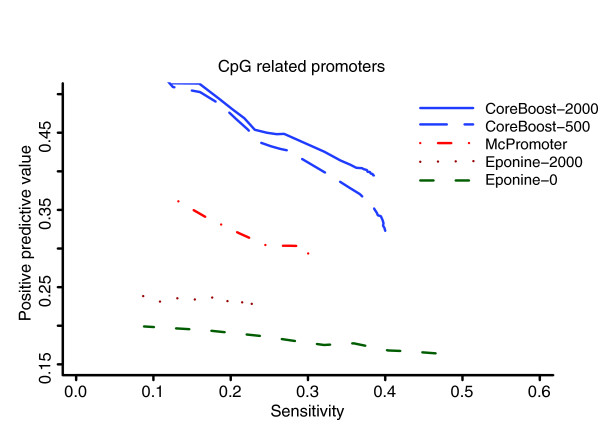
Positive predictive value versus sensitivity for CpG-related promoters. The solid and the long dashed curves are for CoreBoost, with the solid one for the cases clustering predictions within 2,000 bp and the long-dashed one within 500 bp. The dot-dashed curve is for McPromoter which clusters predictions within 2,000 bp as default. The dot and the short-dashed curves are for Eponine, with the dot one for the cases clustering predictions within 2,000 bp and the short-dashed one from the default output of Eponine. bp, base pairs.

Figure 3 shows a plot of PPV versus sensitivity for CpG-related promoters. We see that CoreBoost consistently predicts TSSs better at various thresholds. The threshold achieving about 0.37 sensitivity and 0.37 PPV using 500 bp to cluster predictions is chosen as our default threshold in CoreBoost program for CpG-related promoters. (We choose 500 bp to cluster predictions in CoreBoost in order to find alternative promoters for one gene. The same clustering distance is used in DBTSS.) There are only 85 non-CpG-related test promoters from ChIP-chip data. To achieve an unbiased evaluation of different programs on non-CpG-related promoters, we conducted a study on a larger dataset.

#### Cross-validation for non-CpG-related promoters

**Figure 4:**
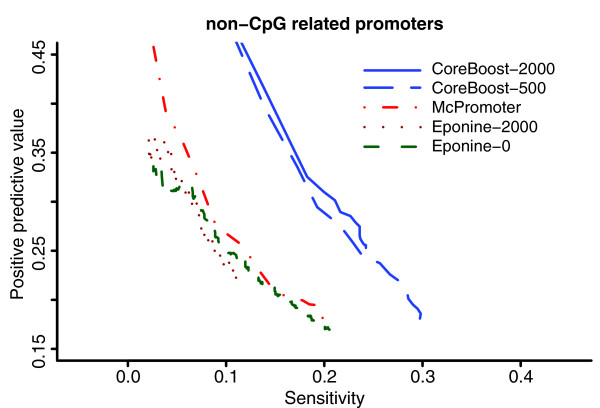
Positive predictive value versus sensitivity for non-CpG-related promoters. The solid and the long-dashed curves are for CoreBoost, with the solid one for the cases clustering predictions within 2,000 bp and the long-dashed one within 500 bp. The dot-dashed curve is for McPromoter, which clusters predictions within 2,000 bp by default. The dot and the short-dashed curves are for Eponine, with the dot one for the cases clustering predictions within 2,000 bp and the short-dashed one from the default output of Eponine. bp, base pairs.

In this section, we report the result of a fivefold cross-validation on a combined set of non-CpG-related promoters from EPD and DBTSS. The set of 299 non-CpG-related promoters from EPD and four-fifths of 1,271 from DBTSS was used to train a model, which was then applied to the remaining one-fifth of the sequences of 2.4 kb each, centered at the DBTSS-annotated TSSs. Figure 4 gives a plot of PPV versus sensitivity comparing CoreBoost, McPromoter, and Eponine. It is evident that CoreBoost consistently outperforms McPromoter and Eponine. The threshold achieving about 0.26 sensitivity and 0.24 PPV using 500 bp to cluster predictions is chosen as the default threshold in the CoreBoost program for non-CpG-related promoters.

### Muscle-specific non-CpG-related promoters

Although we have seen improvement in TSS prediction using CoreBoost, the performance of the computational prediction of TSSs for non-CpG-related promoters remains unsatisfactory. In this section we demonstrate that tissue information can help to improve further the accuracy of prediction. The rationale is that tissue-specific binding sites can act as a guide and, together with the core promoter information, can help in locating the true TSSs.

We trained a more specific classifier based on 84 muscle-specific non-CpG-related promoters, each consisting of 250 bp upstream and 50 bp downstream of the TSS. We used two negative sets, corresponding to two immediate upstream and two immediate downstream segments of 300 bp long, respectively. Leave-one-out cross-validation was carried out for two classifications: one for discriminating promoters from upstream sequences and the other from downstream sequences. We then calculated the sensitivity, PPV, and correlation coefficient (CC). The first classifier (promoter against upstream) gives a sensitivity of 0.70, a PPV of 0.83, and a CC of 0.66, and the second classifier (promoter against downstream) gives a sensitivity of 0.76, a PPV of 0.82, and a CC of 0.69. (For a test segment, if the class probability from LogitBoost is greater than 0.5, then it is classified to Promoter class.) The corresponding *P *values for these CCs are 0.008 and 0.007, respectively, calculated from 1,000 sets of 84 randomly selected non-CpG-related promoters. Even if the size of the training dataset for muscle-specific promoters is much smaller, the CCs are more than 10% greater than those from the fivefold cross-validation of 1,570 non-CpG-related promoters, whose corresponding sensitivity, PPC and CC are 0.64, 0.80, and 0.59 for the first classifier, and 0.66, 0.80, and 0.61 for the second classifier. This indicates that the region [-250,+50] among the muscle-specific promoters does contain additional useful information. Indeed, muscle-specific TFs such as MEF2 and SRF appear among the top features.

## Discussion

We have developed a new core promoter prediction program called CoreBoost, based on two classifiers: one for CpG-related promoters and the other for non-CpG-related promoters. Existing promoter prediction programs have very poor performance on non-CpG-related promoters, and one important contribution of our work is to improve the prediction accuracy for that specific class. We tried to boost the performance in two directions: finding a set of biologically relevant features and applying a robust classification algorithm suitable for class members with heterogenous patterns. We applied our program to Pol II ChIP-chip data and compared it with McPromoter and Eponine, two state-of-the-art TSS finders. The results showed that our program has better accuracy. One nice property of LogitBoost is that it directly provides class probabilities, which are essential for quantifying the confidence of prediction.

Biological systems are complex and hierarchical, and molecular machinery recognizes features at different levels. We believe that genome-scale recognition is at the level of epigenetics and chromatin structure, and fine recognition machinery is subsequently recruited near open chromatin promoter regions to search for TSSs. CoreBoost is designed for and focused on the latter (fine) recognition problem, and thus is not intended for genome-wide searching. In practice, we recommend using some prior information to first identify a search region of about 2.4 kb and then applying CoreBoost. A great deal of prior information is available to focus the search, including the Pol II ChIP-chip data, expressed sequence tag or mRNA alignment, and predicted regions from gene-finding programs such as Genscan [25]. A recent study comparing several promoter predictors on the ENCODE regions of the human genome concluded that the accuracy of promoter prediction can be greatly improved if it is combined with gene prediction [10]. Our results demonstrated that the combined use of ChIP-chip and CoreBoost is able to improve the accuracy of locating TSSs.

We have also described a more specific promoter prediction program based on a set of muscle-specific promoters and demonstrated that, by utilizing tissue information, we can achieve more than 10% better prediction accuracy with much fewer training data. In principle, one could build several specific programs for different tissues, and a grand program consisting of all of these subprograms could be used to predict not only the location of TSSs but also the tissue specificity. Such work has been lacking in the literature and it will be worthwhile to explore this possibility further.

Evidence is accumulating that many Pol II promoters can have multiple TSSs [26]. Some of the predictions not in the close neighborhood of annotated TSSs could eventually turn out to be alternative core promoters. However, there is not enough information or resources to examine all of the false positives at this point. We plan to investigate the possibility of predicting alternative TSSs in the future.

Many of the TSSs that are not CpG-related have changing GC content from upstream to downstream. One recent study [27] showed that considering GC-rich/GC-poor upstream and downstream segments separately yields more biologically meaningful details. We plan to explore whether it may further help to predict promoters via splitting TSSs into subclasses based on the GC content of upstream and downstream segments.

## Materials and methods

### LogitBoost with stumps

Boosting [28-31] has been applied successfully to a wide variety of classification problems. It combines many weak classifiers to boost the performance of a single classifier. Let us denote the training data as (*x*_1_, *y*_1_), ..., (*x*_n_, *y*_n_), which are independently and identically distributed realizations of random variables (*X*, *Y*), where *X *is the feature vector in Rp
 MathType@MTEF@5@5@+=feaafiart1ev1aaatCvAUfeBSjuyZL2yd9gzLbvyNv2Caerbhv2BYDwAHbqedmvETj2BSbqee0evGueE0jxyaibaiKI8=vI8tuQ8FMI8Gi=hEeeu0xXdbba9frFj0=OqFfea0dXdd9vqai=hGuQ8kuc9pgc9s8qqaq=dirpe0xb9q8qiLsFr0=vr0=vr0dc8meaabaqaciGacaGaaeqabaqadeqadaaakeaacaWGsbWaaWbaaSqabeaacaWGWbaaaaaa@3526@, *Y *is the class label from the set {-1,1}, and *n *is the sample size. Denote *f*(*x*) a binary classifier:

f:Rp→{−1,1}.
 MathType@MTEF@5@5@+=feaafiart1ev1aaatCvAUfeBSjuyZL2yd9gzLbvyNv2Caerbhv2BYDwAHbqedmvETj2BSbqee0evGueE0jxyaibaiKI8=vI8tuQ8FMI8Gi=hEeeu0xXdbba9frFj0=OqFfea0dXdd9vqai=hGuQ8kuc9pgc9s8qqaq=dirpe0xb9q8qiLsFr0=vr0=vr0dc8meaabaqaciGacaGaaeqabaqadeqadaaakeaaieGacaWFMbGaaeOoaiaadkfadaahaaWcbeqaaiaadchaaaGccqGHsgIRdaGadaqaaiabgkHiTiaaigdacaGGSaGaaGymaaGaay5Eaiaaw2haaiaac6caaaa@3EC2@

The classifier that minimizes the misclassification risk *P *(*f*(*X*) ≠ *Y*) is called Bayes classifier:

f(X)={1P(Y=1|X)>P(Y=−1|X)−1otherwise.
 MathType@MTEF@5@5@+=feaafiart1ev1aaatCvAUfeBSjuyZL2yd9gzLbvyNv2Caerbhv2BYDwAHbqedmvETj2BSbqee0evGueE0jxyaibaiKI8=vI8tuQ8FMI8Gi=hEeeu0xXdbba9frFj0=OqFfea0dXdd9vqai=hGuQ8kuc9pgc9s8qqaq=dirpe0xb9q8qiLsFr0=vr0=vr0dc8meaabaqaciGacaGaaeqabaqadeqadaaakeaacaWGMbGaaiikaiaadIfacaGGPaGaeyypa0ZaaiqabeaafaqabeGacaaabaGaaGymaaqaaiaadcfacaGGOaGaamywaiabg2da9iaaigdacaGG8bGaamiwaiaacMcacqGH+aGpcaWGqbGaaiikaiaadMfacqGH9aqpcqGHsislcaaIXaGaaiiFaiaadIfacaGGPaaabaGaeyOeI0IaaGymaaqaaiaad+gacaWG0bGaamiAaiaadwgacaWGYbGaam4DaiaadMgacaWGZbGaamyzaiaac6caaaaacaGL7baaaaa@5364@

**Figure 5:**
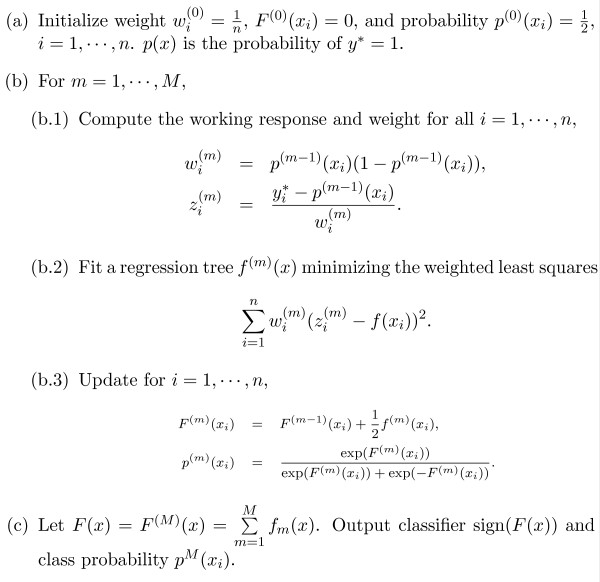
LogitBoost algorithm with trees.

Denote the ensemble of weak classifiers as follows:

F(x)=∑m=1Mcmfm(x),
 MathType@MTEF@5@5@+=feaafiart1ev1aaatCvAUfeBSjuyZL2yd9gzLbvyNv2Caerbhv2BYDwAHbqedmvETj2BSbqee0evGueE0jxyaibaiKI8=vI8tuQ8FMI8Gi=hEeeu0xXdbba9frFj0=OqFfea0dXdd9vqai=hGuQ8kuc9pgc9s8qqaq=dirpe0xb9q8qiLsFr0=vr0=vr0dc8meaabaqaciGacaGaaeqabaqadeqadaaakeaacaWGgbGaaiikaiaadIhacaGGPaGaeyypa0ZaaabCaeaacaWGJbWaaSbaaSqaaiaad2gaaeqaaOGaamOzamaaBaaaleaacaWGTbaabeaakiaacIcacaWG4bGaaiykaaWcbaGaamyBaiabg2da9iaaigdaaeaacaWGnbaaniabggHiLdGccaGGSaaaaa@444E@

where *f*_*m*_(*x*) is the *m*th weak classifier and *c*_*m *_are constants. At each iteration *m*, the observations misclassified at the (*m *- 1)th iteration are given higher weights for the current iteration. The final ensemble is a weighted majority vote of *M *weak classifiers (sign [F(x)]). In this report we use stumps as weak classifiers. A stump is a special type of decision tree [32] with only two terminal nodes. The boosting algorithm sequentially builds a series of stumps, each trained on re-weighted samples. The ensemble of trees has been shown to perform much better than single trees or trees trained independently. For the re-weighting and aggregation, we implement the LogitBoost algorithm [14], which minimizes the negative of binomial log-likelihood as the loss function. This loss function decreases linearly with *yF*(*x*) for misclassified samples and thus is more robust when mislabelled training data are present. The LogitBoost algorithm with decision trees as weak classifiers is outlined in Figure 5. The number *M *of weak classifiers is determined by using cross-validation. Let y* = (*y *+ 1)/2, taking values from {0,1}. LogitBoost directly estimates the posterior class probability:

P(Y=1 | X=x)=exp⁡(F(x))exp⁡(F(x))+exp⁡(−F(x))
 MathType@MTEF@5@5@+=feaafiart1ev1aaatCvAUfeBSjuyZL2yd9gzLbvyNv2Caerbhv2BYDwAHbqedmvETj2BSbqee0evGueE0jxyaibaiKI8=vI8tuQ8FMI8Gi=hEeeu0xXdbba9frFj0=OqFfea0dXdd9vqai=hGuQ8kuc9pgc9s8qqaq=dirpe0xb9q8qiLsFr0=vr0=vr0dc8meaabaqaciGacaGaaeqabaqadeqadaaakeaacaWGqbGaaiikaiaadMfacqGH9aqpcaaIXaGaaeiiaiaabYhacaqGGaGaamiwaiabg2da9iaadIhacaGGPaGaeyypa0ZaaSaaaeaaciGGLbGaaiiEaiaacchacaGGOaGaamOraiaacIcacaWG4bGaaiykaiaacMcaaeaaciGGLbGaaiiEaiaacchacaGGOaGaamOraiaacIcacaWG4bGaaiykaiaacMcacqGHRaWkciGGLbGaaiiEaiaacchacaGGOaGaeyOeI0IaamOraiaacIcacaWG4bGaaiykaiaacMcaaaaaaa@5603@

This is used in the calculation of probability profiles in CoreBoost.

### Multiclass classification using binary classifiers

In our application of LogitBoost to the prediction of promoters, we have three classes: the promoter (P), its immediate upstream sequence (U), and its immediate downstream sequence (D). Instead of the usual way of combining the upstream and downstream sequences into one class, we reduce this three-class problem to two binary ones: one comparing the promoter class against the upstream and the other comparing it against the downstream. The reason is that upstream and downstream sequences are very different from each other. Separate classifiers can pick up the most discriminative features for classifying promoters against upstream or downstream sequences. Let us denote the following as the probability of *Y *belonging to the promoter class based on the binary classifier discriminating promoters from the upstream and from the downstream, respectively:

*p*_1 _= *P*(*Y *∈ *P*|*X*, *P*, *U*) and *p*_2 _= *P*(*Y *∈ *P*|*X*, *P*, *D*)

The probability *p *of *Y *belonging to promoter class in the three-class setting can be calculated as follows:

P(Y∈P|X,P,U,D)=P(Y∈P|X)P(Y∈P|X)+P(Y∈U|X)+P(Y∈D|X)=P(Y∈P|X)(P[Y∈P|X]+P[Y∈U|X])+(P[Y∈D|X]+P[Y∈P|X])−P(Y∈P|X)=P(Y∈P|X)P(Y∈P|X)/P[Y∈P|X,P,U)+P(Y∈P|X)/P(Y∈P|X,P,D)−P(Y∈P|X)=11/p1+1/p2−1=p1p2p1+p2−p1p2.
 MathType@MTEF@5@5@+=feaafiart1ev1aaatCvAUfeBSjuyZL2yd9gzLbvyNv2Caerbhv2BYDwAHbqedmvETj2BSbqee0evGueE0jxyaibaiKI8=vI8tuQ8FMI8Gi=hEeeu0xXdbba9frFj0=OqFfea0dXdd9vqai=hGuQ8kuc9pgc9s8qqaq=dirpe0xb9q8qiLsFr0=vr0=vr0dc8meaabaqaciGacaGaaeqabaqadeqadaaakeaafaqabeqbbaaaaeaacaWGqbGaaiikaiaadMfacqGHiiIZcaWGqbGaaiiFaiaadIfacaGGSaGaamiuaiaacYcacaWGvbGaaiilaiaadseacaGGPaGaeyypa0ZaaSaaaeaacaWGqbGaaiikaiaadMfacqGHiiIZcaWGqbGaaiiFaiaadIfacaGGPaaabaGaamiuaiaacIcacaWGzbGaeyicI4SaamiuaiaacYhacaWGybGaaiykaiabgUcaRiaadcfacaGGOaGaamywaiabgIGiolaadwfacaGG8bGaamiwaiaacMcacqGHRaWkcaWGqbGaaiikaiaadMfacqGHiiIZcaWGebGaaiiFaiaadIfacaGGPaaaaaqaaiabg2da9maalaaabaGaamiuaiaacIcacaWGzbGaeyicI4SaamiuaiaacYhacaWGybGaaiykaaqaaiaacIcacaWGqbGaai4waiaadMfacqGHiiIZcaWGqbGaaiiFaiaadIfacaGGDbGaey4kaSIaamiuaiaacUfacaWGzbGaeyicI4SaamyvaiaacYhacaWGybGaaiyxaiaacMcacqGHRaWkcaGGOaGaamiuaiaacUfacaWGzbGaeyicI4SaamiraiaacYhacaWGybGaaiyxaiabgUcaRiaadcfacaGGBbGaamywaiabgIGiolaadcfacaGG8bGaamiwaiaac2facaGGPaGaeyOeI0IaamiuaiaacIcacaWGzbGaeyicI4SaamiuaiaacYhacaWGybGaaiykaaaaaeaacqGH9aqpdaWcaaqaaiaadcfacaGGOaGaamywaiabgIGiolaadcfacaGG8bGaamiwaiaacMcaaeaacaWGqbGaaiikaiaadMfacqGHiiIZcaWGqbGaaiiFaiaadIfacaGGPaGaai4laiaadcfacaGGBbGaamywaiabgIGiolaadcfacaGG8bGaamiwaiaacYcacaWGqbGaaiilaiaadwfacaGGPaGaey4kaSIaamiuaiaacIcacaWGzbGaeyicI4SaamiuaiaacYhacaWGybGaaiykaiaac+cacaWGqbGaaiikaiaadMfacqGHiiIZcaWGqbGaaiiFaiaadIfacaGGSaGaamiuaiaacYcacaWGebGaaiykaiabgkHiTiaadcfacaGGOaGaamywaiabgIGiolaadcfacaGG8bGaamiwaiaacMcaaaaabaGaeyypa0ZaaSaaaeaacaaIXaaabaGaaGymaiaac+cacaWGWbWaaSbaaSqaaiaaigdaaeqaaOGaey4kaSIaaGymaiaac+cacaWGWbWaaSbaaSqaaiaaikdaaeqaaOGaeyOeI0IaaGymaaaaaeaacqGH9aqpdaWcaaqaaiaadchadaWgaaWcbaGaaGymaaqabaGccaWGWbWaaSbaaSqaaiaaikdaaeqaaaGcbaGaamiCamaaBaaaleaacaaIXaaabeaakiabgUcaRiaadchadaWgaaWcbaGaaGOmaaqabaGccqGHsislcaWGWbWaaSbaaSqaaiaaigdaaeqaaOGaamiCamaaBaaaleaacaaIYaaabeaaaaGccaGGUaaaaaaa@E37A@

Our study shows that better classification accuracy results from use of two binary classifiers rather than one combining the upstream and downstream sequences. (Detailed comparisons are given in Additional data file 1.)

### Datasets

We assign promoter sequences as non-CpG related, if the normalized CpG content of the 3 kb centered at the TSS is less than 0.3, and as CpG related otherwise. (Normalized CpG content was computed as in [13]: *f*_*CG*_/*e*_*CG*_, where *f*_*CG *_is observed CG frequency, *e*_*CG *_is the expected frequency calculated as [(*f*_*C *_+ *f*_*G*_)/2]^2^, and *f*_*C *_and *f*_*G *_are the frequencies of C and G, respectively.) This working definition is based on the observation that the CpG content follows a bimodal distribution, which naturally separates the promoters into two classes [11,13]. Saxonov and coworkers [13] used 0.35 as a threshold to define CpG-related or non-CpG-related promoters. Better classification results are produced when we are more strict in defining non-CpG-related promoters. (Additional data file 1 gives detailed comparisons between the program using 0.35 as the threshold and the program using 0.3 as the threshold.)

To build any promoter model, one needs a collection of promoter sequences with high-quality annotation. We used the annotations from the EPD (version 79) [33] and the DBTSS (version 3.0) [2]. (EPD is based on experimentally determined TSSs, and DBTSS on full-length oligo-capped cDNA sequences.) EPD generally has better quality annotation than DBTSS, but because there are only 299 non-CpG-related promoters from EPD we also included 1,271 DBTSS-annotated promoters for training. After removing redundancy, we had 1,445 CpG-related promoters from EPD and 1,570 non-CpG-related promoters combining both EPD and DBTSS promoters. The promoter sequences 250 bp upstream and 50 bp downstream of the TSS were extracted as the positive training set. The four (two upstream and two downstream) nonoverlapping consecutive segments immediately upstream and downstream of the positive set were used as our negative training set.

In the fivefold cross-validation study for non-CpG-related promoters, the set of 299 non-CpG-related promoters from EPD and four-fifths of 1,271 promoters from DBTSS was used to train a model, and the remaining set of one-fifth of the sequences of 2.4 kb each, centered at the DBTSS-annotated TSS, was used as test data.

For comparison, we also applied different programs to the sequences extracted from the genome-wide mapping data of promoters obtained from the ChIP-chip technology [4]. Testing the trained model on a separate dataset is important to achieve an unbiased evaluation. Therefore, we kept those sequences 2.4 kb centered at the probes where there was only one DBTSS annotation and no more than one probe in the same sequence. This left us with 85 non-CpG-related and 1,765 CpG-related test sequences of 2.4 kb each.

From CSHL Mammalian Promoter Database (CSHLmpd) [34], we defined the tissue-specific activity of each human promoter based on the tissue information of mRNAs and expressed sequence tags overlapping promoters at the 5' end. We found most housekeeping gene promoters to be CpG related, as expected, whereas promoters active in only a few tissues are more likely to be non-CpG related, consistent with previous reports [26,35]. Among 636 so-called tissue-specific non-CpG-related promoters, 84 are muscle specific. We applied leave-one-out cross-validation to this set of 84 promoters.

### CoreBoost features

In this section, we describe the features of CoreBoost in more detail. There are three categories: motif features, including core promoter elements and TFBSs; mechanical properties of promoter DNA; and sequence features from Markovian modeling of promoter sequences and *k*-mer frequencies.

There are 14 features related to core promoter elements. Weight matrices from a previous report [36] were used to calculate the scores of core promoter elements (TATA, Inr, and CCAAT- and GC-box), with the suggested search regions and motif thresholds. For these four elements, pairwise scores were also computed. The score of a pair of core promoter elements is the sum of the scores of each element weighted by the empirical distribution of distances for that pair. Other core promoter elements, such as downstream promoter element, TFIIB recognition element and motif ten element, were searched for in the form of regular expressions [20,37]. A TSS weight matrix was built from the 10 bp segments (-5 to +5 bp) from the training data and was used to score the region from position 246 to 255 of a test segment.

We used 365 vertebrate weight matrices from TRANSFAC [38] to scan DNA sequences with the tool featuretab, which is part of the Comprehensive Regulatory Element Analysis and Discovery (CREAD) suite of sequence analysis tools [39]. The maximal score across a sequence for a TF weight matrix was weighted based on the empirical distribution of positional preference of that TF. The empirical distribution was estimated from the output of MATCH [40] with the thresholds to minimize false positives. The region [-250, +50] was equally split into six bins. The corresponding multinomial distribution of the locations of TFBS in [-250, +50] within these six bins was also estimated and used to calculate the density feature; the average log likelihood score of the locations of TFBSs for each sequence.

From Figure 1, we see that there are two sharp peaks around positions -25 and +1. For each peak of a 10 bp window, a weighted score was calculated with weights from the empirical score distribution. The difference between the weighted score of a peak and the average score of its surroundings, as well as the average score of the promoter region, were also used. To capture the characteristic large-scale shapes of the energy/flexibility profiles around TSSs, we also calculated the correlation between a vector of smoothed energy/flexibility scores of a test sequence and the average energy/flexibility profiles up to 250 bp, 500 bp, and 1300 bp around TSSs. We chose 5, 150, and 500 as the length of smoothing window. There are 14 features in this category.

Homogeneous third order Markov models were estimated from the upstream, promoter, and downstream sequences. The log-likelihood ratios between promoter and upstream, and between promoter and downstream were used as features. The frequencies of 1-mers or 2-mers related to C or G were also calculated. There are six features in this category.

### CoreBoost web interface

We have constructed a CoreBoost web interface [41]. It takes an input sequence and chooses either the CpG-related or non-CpG-related program based on the calculated normalized GC score. The program works by sliding a window of 300 bp along the input sequence. Because of the use of large-scale features, the current version requires users to input 1.3 kb flanking sequences together with their interested searching segment. Only the positions 1.3 kb from the start and before the end are searched for putative TSSs. Positions with probability exceeding a prespecified threshold are considered possible candidates. The candidates within 500 bp are clustered and the one with the best score is output as the putative TSS. The default thresholds are set to achieve 0.37 sensitivity and 0.37 PPV for CpG-related promoters, and 0.26 sensitivity and 0.24 PPV for non-CpG-related promoters. Users can also choose to search the negative strand as well, in which case the prediction with the best score within 500 bp on the negative strand is also output.

### Performance measures

Sensitivity, PPV, and CC are defined as follows:

Sensitivity=TPTP+FNPPV=TPTP+FPCC=TP×TN−FP×FN(TP+FP)(TP+FN)(TN+FP)(TN+FN),
 MathType@MTEF@5@5@+=feaafiart1ev1aaatCvAUfeBSjuyZL2yd9gzLbvyNv2Caerbhv2BYDwAHbqedmvETj2BSbqee0evGueE0jxyaibaiKI8=vI8tuQ8FMI8Gi=hEeeu0xXdbba9frFj0=OqFfea0dXdd9vqai=hGuQ8kuc9pgc9s8qqaq=dirpe0xb9q8qiLsFr0=vr0=vr0dc8meaabaqaciGacaGaaeqabaqadeqadaaakeaafaqabeWabaaabaGaam4uaiaadwgacaWGUbGaam4CaiaadMgacaWG0bGaamyAaiaadAhacaWGPbGaamiDaiaadMhacqGH9aqpdaWcaaqaaiaadsfacaWGqbaabaGaamivaiaadcfacqGHRaWkcaWGgbGaamOtaaaaaeaacaWGqbGaamiuaiaadAfacqGH9aqpdaWcaaqaaiaadsfacaWGqbaabaGaamivaiaadcfacqGHRaWkcaWGgbGaamiuaaaaaeaacaWGdbGaam4qaiabg2da9maalaaabaGaamivaiaadcfacqGHxdaTcaWGubGaamOtaiabgkHiTiaadAeacaWGqbGaey41aqRaamOraiaad6eaaeaadaGcaaqaaiaacIcacaWGubGaamiuaiabgUcaRiaadAeacaWGqbGaaiykaiaacIcacaWGubGaamiuaiabgUcaRiaadAeacaWGobGaaiykaiaacIcacaWGubGaamOtaiabgUcaRiaadAeacaWGqbGaaiykaiaacIcacaWGubGaamOtaiabgUcaRiaadAeacaWGobGaaiykaaWcbeaaaaGccaGGSaaaaaaa@7353@

where *TP *stands for true positives, *TN *for true negatives, *FP *for false positives, and *FN *for false negatives.

## Additional data files

The following additional data are available with the online version of this article. Additional data file 1 provides a comparison between the program based on two binary classifiers and that based on one binary classifier, and a comparison between the program using 0.35 as threshold to define CpG-related or non-CpG-related promoters and that using 0.3 as threshold.

## Supplementary Material

Additional data file 1 Shown are a comparison between the program based on two binary classifiers and that based on one binary classifier, and a comparison between the program using 0.35 as threshold to define CpG-related or non-CpG-related promoters and that using 0.3 as threshold. Click here for file

## Figures and Tables

**Table 1:** Input features to train CoreBoost

Feature list	Details
Core promoter elements	Score of each core element Weighted score of pairs between TATA, Inr, and CCAAT- and GC-box
TFBSs	Weighted maximal scores for weight matrices from TRANSFAC and density of TFBS
Mechanical properties	Weighted energy/flexibility scores around position -25 and +1 Average energy/flexibility scores Correlation with the empirical average energy/flexibility profile
Markovian score	Likelihood ratios from homogeneous third order Markov models
k-mer frequency	Frequency of 1- or 2-mers related to nucleotide G or C

TFBS, transcription factor binding site.

**Table 2:** Top features in CoreBoost

Classifier type		Features
CpG	P versus U	Log-likelihood ratios from third order Markov chain, log-likelihood ratios from TSS weight matrix
		GC-box score, weighted score of transcription factor NFY, weighted energy score at position +1
		Weighted score of transcription factor YY1, TATA score, weighted score of transcription factor ELK1
		MTE score, weighted score of transcription factor CREB
	P versus D	Log-likelihood ratios from third order Markov chain, GC-box score
		Weighted score of transcription factor NFY
		Log-likelihood ratios from TSS weight matrix
		Difference between the energy score around positions -25 and +1 and the average from surroundings
		Log-likelihood ratios from transcription factor ELK1, frequency of G+C
		Log-likelihood ratios from transcription factor YY1, TATA score, frequency of G
Non-CpG	P versus U	Correlation between vector of energy scores and empirical average energy profile
		Log-likelihood ratios from third order Markov chain, TATA score
		Difference between the energy score around positions -25 and +1 and the average from surroundings
		Weighted energy at position +1
		Proportion of Inr and GC-box pair within 10 bp of observed distance, Inr score.
	P versus D	Correlation between vector of energy scores and empirical average energy profile, TATA score
		Log-likelihood ratios from third order Markov chain
		Weighted energy at position +1
		Correlation between vector of flexibility scores and empirical average flexibility profile, Inr score
		Difference between the flexibility score around position +1 and the average from surroundings, GC-box score

bp, base pairs; D, immediate downstream sequence; P, promoter; TSS, transcription start site; U, immediate upstream sequence.
