# Supplementary material for: Boosting with stumps for predicting transcription start sites
Source: Genome Biol. 2007 Feb 2;8(2):R17. doi: 10.1186/gb-2007-8-2-r17 (PMC1852414; doi:10.1186/gb-2007-8-2-r17)
Supplement: Additional data file 1 — Shown are a comparison between the program based on two binary classifiers and that based on one binary classifier, and a comparison between the program using 0.35 as threshold to define CpG-related or non-CpG-related promoters and that using 0.3 as threshold. [file gb-2007-8-2-r17-S1.pdf]

Supplementary material for "Boosting with stumps for predicting transcription start sites"

Xiaoyue Zhao, Zhenyu Xuan, and Michael Q. Zhang

In this supplementary material, we compared the current CoreBoost program with two new programs on another independent test data. We trained two new programs: one was based on a single binary classifier combining the upstream and downstream into one negative set using 3,015 promoters (1,445 CpG related promoters and 1,570 non-CpG related promoters); the other one was based on two binary classifiers with 3,483 promoters using 0.35 as threshold to split the promoters (1,312 with normalized GC score >= 0.35 and 2,171 with normalized GC score < 0.35). We selected 5,000 promoters which have the most 5'-EST evidence from recently released DBTSS (version 5.0) (the training and test data used in the manuscript was based on EPD and DBTSS version 3.0). After removing the promoters which are within 2.4kb of the promoters already used in the manuscript, we had 1,599 promoters (including 213 non-CpG related promoters based on threshold 0.3) as another independent test data. Figure 1 gives the comparison between the CoreBoost program and the program based on a single binary classifier. Figure 2 gives the comparison between the CoreBoost program and the program using 0.35 as threshold to define CpG and non-CpG related promoters. We can see that the current CoreBoost program based on two binary classifiers and using 0.3 as threshold is better than the programs based on one binary classifier and using 0.35 as threshold.

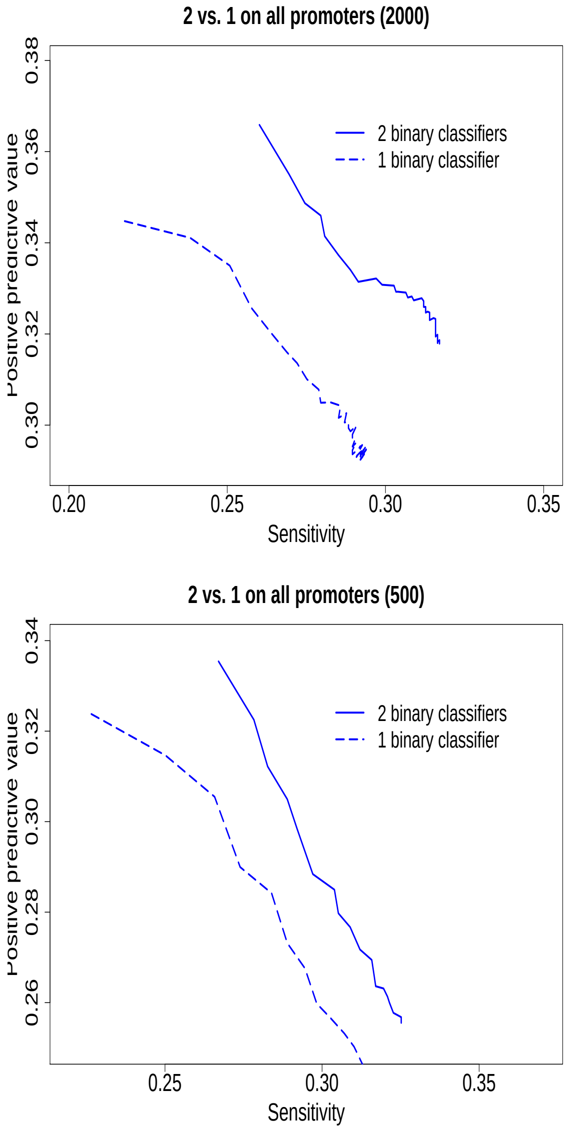

Figure 1: Comparison between the current CoreBoost program based on two binary classifiers and the program based on a single binary classifier. The solid curves are for the CoreBoost program and the long-dashed curves are for the program based on a single binary classifier, with the top one for the cases clustering predictions within 2000bp and the bottom one within 500bp.

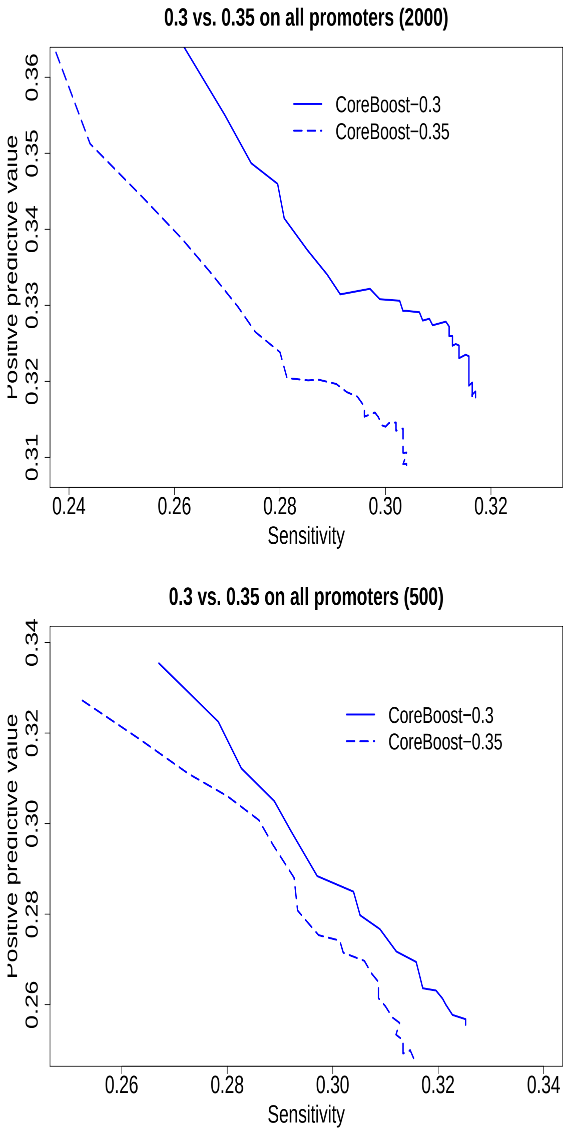

Figure 2: Comparison between the current CoreBoost program using 0.3 as threshold and the program using 0.35 as threshold. The solid curves are for the CoreBoost program and the long-dashed curves are for the program using 0.35 as threshold, with the top one for the cases clustering predictions within 2000bp and the bottom one within 500bp.
